# Study protocol for a prospective, randomized, multicenter trial to investigate the influence of peripheral nerve stimulation on patients with chronic sacroiliac joint syndrome (SILENCING)

**DOI:** 10.1186/s13063-024-08067-z

**Published:** 2024-03-28

**Authors:** Tarik Alp Sargut, Dimitri Tkatschenko, Anton Früh, Jochen Tüttenberg, Alexander Heckert, Steffen Fleck, Anja Kuckuck, Simon Heinrich Bayerl

**Affiliations:** 1 Department of Neurosurgery, Charité—Universitätsmedizin Berlin, Charitéplatz 1, 10117 Berlin, Germany; 2 Department of Neurosurgery, Clinical Center Idar-Oberstein, 55743 Idar-Oberstein, Germany; 3 Department of Neurosurgery, University of Oldenburg, Oldenburg, Germany; 4 Department of Neurosurgery, University Medicine Greifswald, Sauerbruchstrasse, Greifswald, Germany

**Keywords:** Sacroilicac joint pain, Neuromodulation, Peripheral nerve stimulation, Randomized controlled trial

## Abstract

**Background:**

The prevalence of sacroiliac joint pain (SIJP) is estimated to be 10–30% in patients with chronic low back pain. Numerous conservative and surgical treatment modalities for SIJP have been described with limited evidence regarding long-term pain relief.

Spinal cord stimulation (SCS) is a well-established technique to treat patients with chronic low back pain. However, the effect on patients with SIJP is not consistent. Therefore, peripheral nerve stimulation (PNS) for chronic SIJP was implemented in experimental trials. Clinical data on PNS for SIJP is still lacking. The authors present a case series and a protocol for a prospective, multicenter study to determine the effect of PNS in patients with chronic intractable SIJP.

**Method:**

A multicenter, prospective randomized controlled trial was designed. Patients with chronic intractable SIJP will be recruited and randomized in a 4:3 ratio to either the peripheral nerve stimulation group or to the best medical treatment group. A total of 90 patients are planned to be enrolled (52 in the PNS group and 38 in the BMT group). Patients in the intervention group receive a percutaneous implantation of a unilateral or bilateral lead which is externalized for a trial phase for 3–14 days. After trial phase only patients with at least 50% reduction of pain receive an impulse generator for permanent stimulation. Regular visits for participants are planned on day 0, after 3 months (± 30 days), 6 months (± 30 days), and 12 months (± 60 days). The primary outcome measurements is the difference in Numeric Pain Rating Scale (NRS) between baseline and after 6 months. Secondary outcomes is improvement of pain associated disability (ODI) and improvement of health-related quality of life after 6 and 12 months.

**Discussion:**

We have described the protocol for a prospective, multicenter, randomized trial evaluating the influence of PNS on patients with chronic sacroiliac joint syndrome. We believe that PNS on patients with chronic sacroiliac joint syndrome will show promising results regarding pain relief and quality of life in comparison to BMT after 12 months. The design of this trial promises high evidence in comparison to the data to date.

**Trial registration:**

ClinicalTrials.gov, NCT05357300. Registered on April 26, 2022.

## Administrative information

**Table:** 

Title {1}	Study protocol for a prospective, randomized, multicenter trial to investigate the influence of peripheral nerve stimulation on patients with chronic sacroiliac joint syndrome (SILENCING)
Trial registration {2a and 2b}	ClinicalTrials.gov Identifier: NCT05357300, registered April 26, 2022; https://www.clinicaltrials.gov/ct2/show/NCT05357300
Protocol version {3}	SILENCING protocol v1, May 2, 2022
Funding {4}	Boston Scientific Corporation (Vestatstraat 6, 6468 EX Kerkrade, Netherlands). The funders had no role in study design, data collection, or preparation of the manuscript
Author details {5a}	*Tarik Alp Sargut,MD* ^*1*^ *;Dimitri Tkatschenko, MD* ^*1*^ *; Anton Früh, MD* ^*1*^ *; Jochen Tüttenberg, MD* ^*2*^ *; Alexander Heckert, MD* ^*3*^ *; Steffen Fleck, MD* ^*4*^ *; Anja Kuckuck* ^*1*^ *;Simon Heinrich-Bayerl, MD* ^*1*^ ^1^Department of Neurosurgery, Charité – Universitätsmedizin Berlin, Berlin, Germany ^2^Department of Neurosurgery, Clinical Center Idar-Oberstein, 55,743, Idar-Oberstein, Germany ^3^Department of Neurosurgery, University of Oldenburg, Oldenburg, Germany ^4^Department of Neurosurgery, University Medicine Greifswald, Sauerbruchstrasse, Greifswald, Germany
Name and contact information for the trial sponsor {5b}	Charité – Universitätsmedizin Berlin Department of Neurosurgery Hindenburgdamm 30 10,203 Berlin, Germany simon.bayerl@charite.de
Role of sponsor {5c}	The study sponsor as initiating study site has implemented the study design, data collection, management, analysis and interpretation of data. Preparation of the manuscript and the decision to submit was made by the sponsor. The funders had no role or authority in study design, data collection, management, analysis, or interpretation of data. They will have no role in the writing of associated publications and the decision to submit papers for publication

### Introduction

#### Background and rationale {6a}

The prevalence of sacroiliac joint pain (SIJP) is estimated to be between 10 and 30% in patients with chronic low back pain, causing up to 4% of total working force loss in Germany [1, 2]. As the socioeconomic impact remains huge, numerous conservative and surgical treatment modalities for SIJP have been described.

Initial management of patients with physiotherapy and best medical treatment is complemented by intra-articular injections, radiofrequency or cryo-ablation of the lateral branches of S1 to S3, and sacroiliac joint fusion. To date, evidence regarding long-term pain relief remains limited [3–7]. Spinal cord stimulation (SCS) is a well-established technique to treat patients with chronic low back pain [8–11]. However, the effect on patients with SIJP is not consistent. Therefore, peripheral nerve stimulation for chronic SIJP was implemented in experimental trials. Clinical data on peripheral nerve stimulation (PNS) for SIJP is still lacking as only limited retrospective data and case reports have been published [1, 12]. Therefore, the authors present a protocol for a prospective, multicenter randomized study to determine the effect of PNS in patients with chronic intractable SIJP.

## Objectives {7}

The primary objective of this study is to evaluate the influence of peripheral nerve stimulation on the pain level, pain-associated disability and quality of life in patients with chronic intractable SIJP with a high evidence level. It is hypothesized that PNS of the rami dorsales (L5–S3) in patients with chronic sacroiliac joint pain is superior compared to the best medical treatment 6 months after therapy.

## Trial design {8}

This is an investigator initiated prospective, randomized, multicenter trial to investigate the influence of peripheral nerve stimulation on patients with chronic sacroiliac joint syndrome. Patients with chronic intractable SIJ pain will be recruited. After informed consent, the patients will be randomized 4:3:

1. PNS group—peripheral nerve stimulation.
2. BMT group—best medical treatment + physiotherapy (current gold standard).

After 6 months, patients from BTM-group with not satisfactory outcome will be offered to switch to the PNS therapy.

## Methods: participants, interventions and outcomes

### Study setting {9}

Participants will be recruited from outpatient clinics of academic hospitals and community clinics all over Germany. The Charité Coordination Clinical Trial Office (CTO) is helping with study planning and is performing the monitoring and the digital data management of the study. Electronic CRFs were created together with the CTO. Data will be collected according to the study schedule. List of study sites can be obtained at CTO.

### Eligibility criteria {10}

#### Inclusion criteria

- Patients with chronic sacroiliac joint pain refractory to conservative treatment.
- Patients with a Numeric Rating Scale (NRS) score of at least 60/100.
- Patients with temporary pain reduction of at least 50% (NRS) after fluoroscopy guided SIJ infiltration
- Patients received conservative treatment for at least 6 months including physiotherapy and pain medication.
- Patients which from a medical point of view have enough cognitive capacities to understand the programming of Implantable Pulse Generators (IPGs).

#### Exclusion criteria

- Patient’s age < 18 years.
- Pregnancy.
- Acute traumatic injury of the SIJ.
- Active inflammation or neoplastic infiltration of the SIJ.
- Neoplastic diseases of the spine.
- Neoplastic diseases of the spine.
- Spinal surgery within the last 6 months.
- The SIJ pain is not the leading symptom.
-  Contraindication for neuromodulation device (severe psychiatric disease, severe coagulation disorder, acute infection, active autoimmune disease with immunosuppression).

All participating centers require spinal imaging and blood samples as a standard prior to treatment of SIJP to rule out other pathologies as cause of the pain.

### Who will take informed consent? {26a}

Written informed consent from potential trial participants will be obtained from the investigators at each clinic. As potential participants are seen in specialized neuromodulation outpatient clinics, patients meeting eligibility criteria are informed in person by clinicians and provided information material.

### Additional consent provisions for collection and use of participant data and biological specimens {26b}

Biological specimens will not be collected by this trial and therefore not stored or used for research. All data collection processes are provided by the informed consent form. Collected data will be de-identified and not used for other purpose.

## Interventions

### Explanation for the choice of comparators {6b}

Since evidence regarding long-term pain relief with the different treatment modalities such as intra-articular injections, radiofrequency ablation, cryoablation and sacroiliac joint fusion is limited, the gold standard remains best medical treatment (BMT) combined with physiotherapy. Therefore, this trial compares outcomes of PNS of the rami dorsales (L5–S3) to outcomes of BMT and physiotherapy. BMT will be performed at a specialized outpatient clinic to ensure high quality level of treatment. On top of that, physiotherapy has to be done continuously for at least 2 months during this period. Patient will receive the prescription from the study centers to ensure implementation.

### Intervention description {11a}

**Fig. 1:**
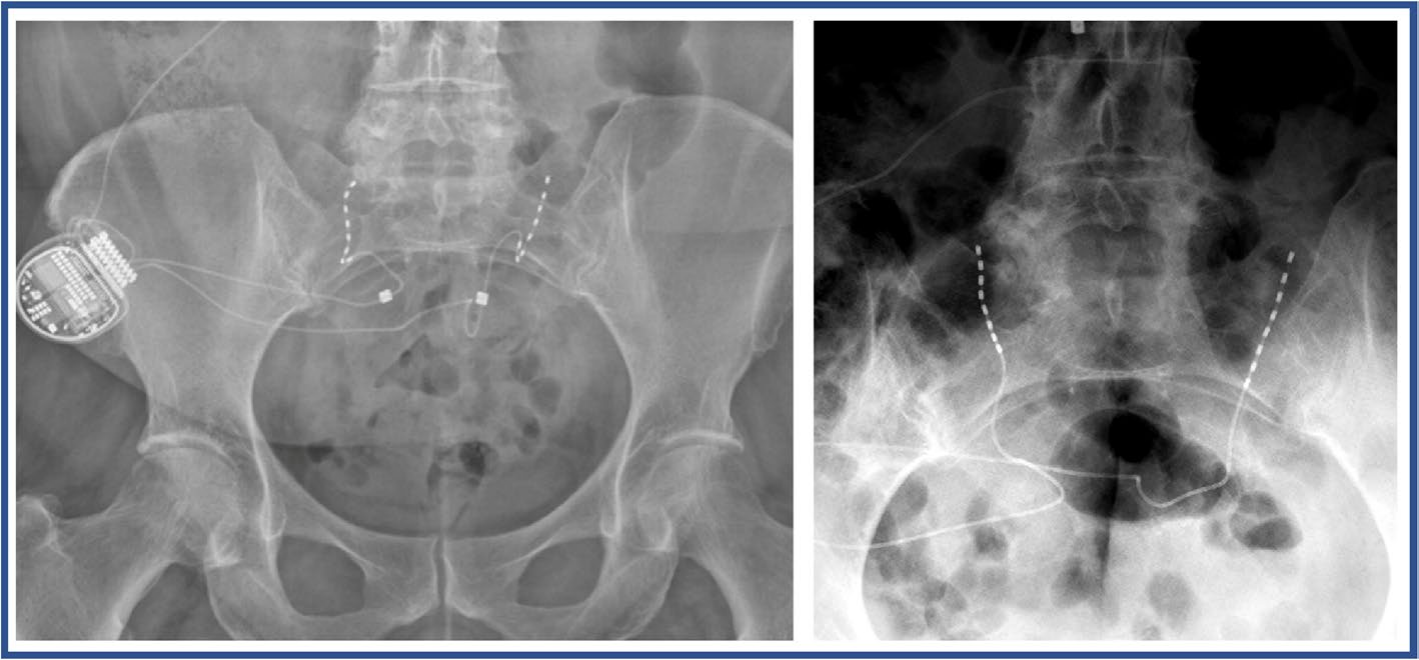
Postoperative coronar X-rays of two different patients with placement of the leads between the sacroiliac joint and the sacral foramina. Note that the Implantable Pulse Generator in the gluteal pocket is displayed on the right

**Fig. 2:**
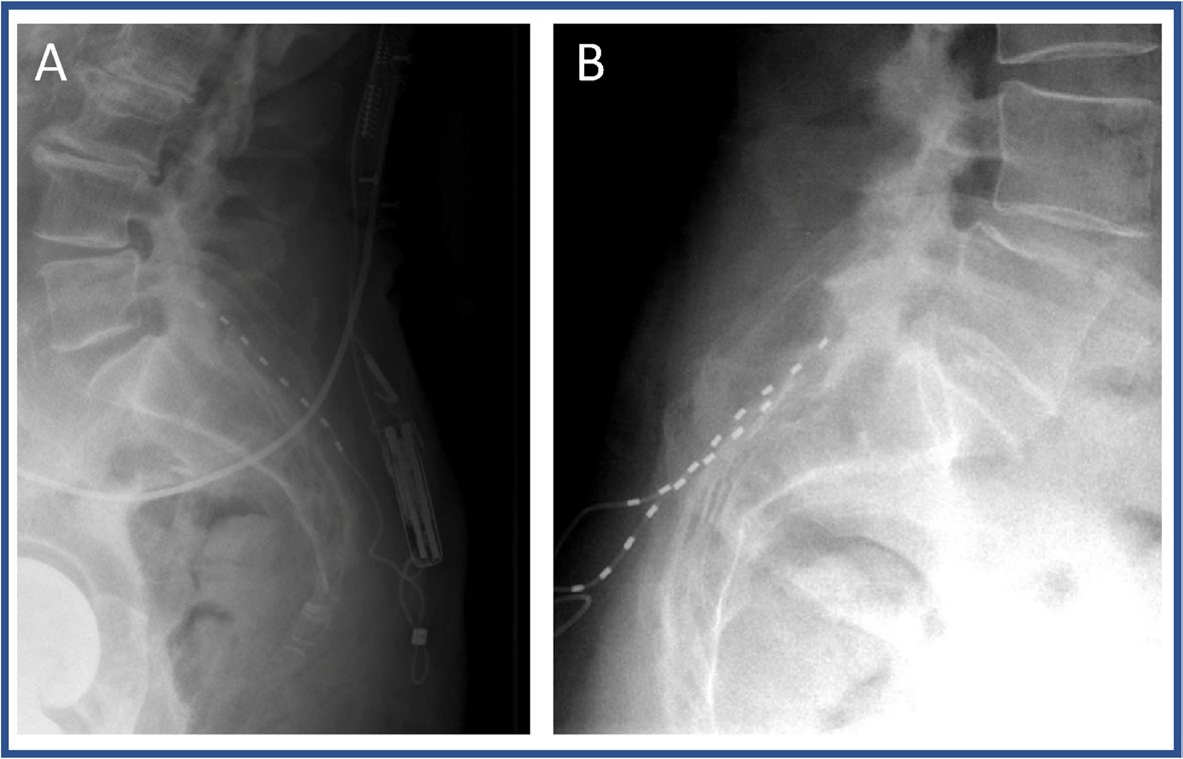
Postoperative sagittal X-rays of two different patients with placement of the leads close to the sacral surface. **A** Patient with unilateral lead. **B** Patient with bilateral leads

The operative technique of PNS of the rami dorsales L5–S3 has been described previously [1]. Surgery can be performed in local anaesthesia with additional sedation or in general anaesthesia. Patients are placed in prone position with the head lying in a soft head shell or pillow. The sacroiliac joint is marked on both sides and the skin is disinfected with Octeniderm (Schülke & Mayr GmbH, Norderstedt, Germany) in usual fashion. The skin and the periost of the sacrum medial to the SIJ is infiltrated with 5–10 ml of 1% lidocaine, if patients did not receive general anaesthesia. After 2-cm skin incision and subcutaneous preparation, a 14-gauge hollow hypodermic needle (Entrada™, Needle, Boston Scientific, MA, USA) is placed along the sacrum lateral to the dorsal foramen and medial to the SIJ with under fluoroscopic guidance and the stylet is removed. The percutaneous PNS lead (8-contact, Linear™ 3–6 lead, Boston Scientific, MA, USA) is inserted through the needle and positioned between the sacroiliac joint and the sacral foramina as close to the sacral surface as possible with contacts stimulation the rami dorsales S1-S3 (Fig. 1). To verify optimal placement, a lateral fluoroscopic scan is performed (Fig. 2). The lead is fixed with an anchor (BostonScientific, MA, USA) and can be tunneled to a separately prepared subcutaneous gluteal pocket. Here it will be connected to an extension lead, which will be tunneled through the skin to enable external stimulation.

The part of the extension lead protruding the skin is sutured sturdily and connected to the external stimulator. After successful trial phase of 3–14 days patients with at least 50% pain reduction in NRS score, patients receive the implantation of the impulse generator (WaveWriter Alpha™, Boston Scientific, MA, USA), which will be connected to the stimulation lead for permanent stimulation. The extension will be discarded. Therefore, the extension lead is cut close to the skin and removed. The impulse generator is then inserted subcutaneously into the initially prepared subcutaneous pocket. Therefore, the skin incision is widened to a 5–6-cm skin incision, the impulse generator is fixed with a suture and the generator is connected to the lead.

If patients did not experience a 50% pain relief, the leads will be explanted.

### Criteria for discontinuing or modifying allocated interventions {11b}

As study participation is voluntary, all participants may withdraw from the study at any time. Patients randomized to the PNS group can have their PNS system explanted at any time if they want to withdraw from the study. The study team will not collect any further data from participants who withdraw. Data already collected will be retained, as agreed in written informed consent. After withdrawal from the study, participants will not be allowed to enter back into the study at a later date.

### Strategies to improve adherence to interventions {11c}

Participants are able to contact a study nurse via mail or phone call at any time for questions or technical support with the PNS system. Additionally, study investigators are contacting the participants at each follow-up visit and examine wound healing and stimulation settings together with the technicians from Boston Scientific.

### Relevant concomitant care permitted or prohibited during the trial {11d}

Concomitant intervention for sacroiliac joint pain is prohibited during the trial. This applies for both study groups. After randomization, it will not be allowed to receive other treatment modalities such as intraarticular injections, radiofrequency ablation, cryoablation or sacroiliac joint fusion.

### Provisions for post-trial care {30}

Participants with PNS are regularly seen at neuromodulation outpatient clinic in post-trial care.

There is no anticipated harm and compensation for trial participation. No provision for post-trial care will not be conducted.

### Outcomes {12}

The primary outcome is the difference in Numeric Pain Rating Scale (NRS) between baseline and after 6 months.

#### Secondary outcomes

1. Proportion of patients with ≥ 50% reduction in sacroiliac joint pain (responder) on the NRS (0–100) at 6 and 12 months.
2. Improvement of pain associated disability (ODI) after 6 and 12 months.
3. Improvement of Health-related quality of life after 6 and 12 months.
4. Difference in Numeric Pain Rating Scale (NRS) between baseline and after 12 months.

Clinical relevance and standardization of NRS and ODI has been validated previously. The majority of clinical trials on PNS are monitoring outcomes by NRS and ODI (15–18).

Health economics outcomes:

1. Ability to work, if patients were unable to work because of the chronic back pain
2. Doctor visits after inclusion in the study

## Participant timeline {13}

Participants will participate in the study for 12 months.

1. All patients in the BMT group receive best medical treatment for chronic back pain in the outpatient department and receive physiotherapy treatment for at least 2 months.
2. Patients in the PNS group receive the following procedure:
  1. Patients will be prepared for the surgical procedure with blood sample, informed consent and potentially further diagnostic procedures.
  2. The surgery will be performed with analgesic sedation or in general anaesthesia:

Percutaneous implantation of the lead (unilateral/bilateral). Connection to extension and externalization

Trial phase for 3–14 days with NRS monitoring.

After trial phase, only patients with at least 50% reduction of pain (NRS) receive the impulse generator gluteal/abdominal for permanent stimulation.

The study schedule is presented in Table 1.

**Table 1:** Study schedule

	Screening	V1 (day 1)	V2 (after trial)	V3 (3 months ± 30 days)	V4 (6 months ± 30 days)	V5 (12 months ± 60 days)	Time expenditure (min.)
NRS (0–10) (1 item)	x	x	x	x	x	x	6 × 10 60
ODI (10 items)		x		x	x	x	4 × 20 80
SF-36 (36 items)		x		x	x	x	4 × 30 120
ADS-K		x		x	x	x	4 × 30 120
Patient satisfaction with pain relief (0–10) (1 item)		x		x	x	x	4 × 10 40
PSQI (24 items)		x		x	x	x	4 × 20 80
Neurological status (50 items)		x	x	x	x	x	5 × 30 150
IPG-data (15 items)			x	x	x	x	4 × 30 120

*NRS* Numeric Pain Rating Scale, *ODI* Oswestry Disability Index, *SF-36* The Short Form (36) Health Survey, *ADS-K* General Depression Scale, *PSQI* Pittsburgh Sleep Quality Index, *IPG* implantable pulse generators

## Sample size {14}

Based on our own preliminary data of 10 patients, a mean decrease in NRS score of 3.5 points (standard deviation = 3.1) was observed in the PNS group, whereas the reduction in the BMT group was 1.4 points (standard deviation = 1.7). Childs et al. have previously reported a 2-point difference on the NRS to be clinically meaningful in a multicenter, randomized trial [13].

To detect the observed difference (2.1 points in reduction in NRS score) with an estimated standard deviation of 3, a two-sided error of 5% with a power of 80% using an unpaired *t*-test, a case number of 34 per group is needed. Thus, taking into account the 25% non-responders in the PNS group, one would need 46 patients in the intervention group and use a randomization of 4:3 accordingly. Adding 10% additional dropouts, one would need to include a total of 90 patients (52 in the PNS group and 38 in the BMT group) in the study. The sample size planning was performed using nQuery version 7.0. The planning in this study was performed by the study sponsor in collaboration with the local Institute of Biometry and Clinical Epidemiology.

## Role of sponsor {5c}

This is an investigator-initiated study. The sponsor as initiating study site has implemented the study design, data collection, management, analysis and interpretation of data. Preparation of the manuscript and the decision to submit was made by the sponsor.

## Recruitment {15}

Many patients with back pain and specifically sacroiliac joint pain are assigned to specialized outpatient clinics with focus on neuromodulation once a week. The study team is always part of the neuromodulation outpatient clinics, so that patients meeting eligibility criteria are immediately in contact with the investigators. The research coordinator will provide information material describing the study as well as sacroiliac PNS models.

After detailed verbal information, the participants are given a sufficient cooling off period and the opportunity for open questions afterwards. Undecided patients are able to contact a study nurse, making participation on a later time possible. Patients who provide written informed consent to participate in the study will be randomized as described before.

## Patient and public involvement

Although this study might be from relevant public interest due to the high prevalence of SIJP and impact on total working force loss in Germany, there was no patient or public involvement in the design of the study protocol.

## Assignment of interventions: allocation

### Sequence generation {16a}

Participants will be randomized in a 4:3 ratio to either to the PNS group or BMT group (current gold standard).

Randomization is performed by the study team using computer-generated randomization lists with variable block sizes. No stratification is used.

### Concealment mechanism {16b}

Allocation sequence is performed electronically using RedCap prior to the randomization of the participants. After randomization, further concealment is not possible due to the nature of the study.

### Implementation {16c}

Enrollment of participants is done by the investigator or study coordinator. Assignment to intervention is determined by the randomization group. The allocation sequence is performed electronically as described previously. Allocation is therefore not predictable for the study team before randomization.

## Assignment of interventions: blinding

### Who will be blinded {17a}

As patients will be aware of the allocated study arm after randomization, blinding is not possible.

### Procedure for unblinding if needed {17b}

Does not apply.

## Data collection and management

### Plans for assessment and collection of outcomes {18a}

Regular visits for participants are planned on day 0, after 3 months (± 30 days), 6 months (± 30 days) and 12 months (± 60 days). Additionally, patients allocated to the PNS group are evaluated after the trial phase.

The primary and secondary outcome measurements will be acquired by the study group on the visits as described in the study schedule in Table 1. Additionally, ODI (Oswestry Disability Index), NRS (Numeric Pain Rating Scale), SF-36 (The Short Form (36) Health Survey), PSQI (Pittsburgh Sleep Quality Index), ADS-K CES-D (Center of Epidemiological Studies Depression Scale), IPG data and neurological status will be assessed at all scheduled visits.

### Plans to promote participant retention and complete follow-up {18b}

The study group will contact participants by mail and phone throughout the trial and arrange all appointments with participation in this study. The reported scope of 30–60 days in the 3-, 6- and 12-month outpatient visits gives more flexibility to the participants. The importance of the visits is explained to participants in advance. Participants have the opportunity to contact the study nurse at any time for questions concerning follow-up. If participants want to withdraw from the study, a final outpatient visit is scheduled in order to complete data collection.

### Data management {19}

All data are stored in an encrypted form in the REDCap (Research Electronic Data Capture) database. IPG data are recorded by the technical assistants and transferred to the same database. Password protection ensures that only authorized persons from the study have access to participants data. Data collection will be done in the outpatient visits, so that there will be no need for questionnaires by post or mail.

### Confidentiality {27}

All collected personal and medical data will be coded and recorded by the rules of good clinical practice. Disclosure to others than the study team is prohibited. Participants will receive a unique study ID so that data will be stored anonymously. As described previously, the database is secured with a password. Only the steering committee and the study team members when this is necessary in the interest of the trial will have access to the anonymized data.

### Plans for collection, laboratory evaluation and storage of biological specimens for genetic or molecular analysis in this trial/future use {33}

Storage of biological specimens is not conducted in this trial.

## Statistical methods

### Statistical methods for primary and secondary outcomes {20a}

The primary endpoint will be analysed by mixed linear regression with the change in NRS score from baseline to 6 [12] months as dependent and treatment and baseline value as independent variables. Study center will be added as a random intercept. In order to adjust for relevant covariables, a maximum of four additional variables are included in the model due to the sample size limitations. By this procedure, depending on the correlation of the independent variables with the dependent variable, a gain in power is obtained compared with the calculated sample size [14]. Even in the worst case and a correlation of 0, however, at least the power as specified in the sample size calculation is achieved. As a further analysis to investigate time trends, we will apply a linear mixed effects models as before but consider all time points and include timepoint as an additional, discrete factor. Further, the interaction between treatment and timepoint and a random intercept for the patients will be added.

A chi-square test is used to analyse the proportion of patients with at least a 50% reduction in the NRS value. In a second step, if there are enough patients with a corresponding reduction, a mixed logistic regression model with additional covariates and study center as random intercept is created. The secondary endpoints will be analysed analogously to the primary endpoint. All analyses including *p*-values are to be considered purely exploratory. All results will be reported with 95% CIs.

### Interim analyses {21b}

Interim analysis of this trial is not planned.

### Methods for additional analyses (e.g. subgroup analyses) {20b}

Not applicable, no subgroup analysis will be performed.

### Methods in analysis to handle protocol non-adherence and any statistical methods to handle missing data {20c}

Missing data will be avoided as possible by early planning of scheduled visits. If participants are not able to present at the study center, a home visit will be offered. The previously reported guidelines on handling missing data will be followed [15]. All outcome measurements will be collected during the same scheduled visits (Table 1). Missing data of a whole visit will not result in dropouts.

### Plans to give access to the full protocol, participant level-data and statistical code {31c}

The datasets analysed during the current study and statistical code are available from the corresponding author on reasonable request, as is the full protocol. With the publication of the main manuscript, de-identified participant data will be available to promote open science.

## Oversight and monitoring

### Composition of the coordinating center and trial steering committee {5d}

This trial is performed as multicenter study. The coordinating center group is composed of the principal investigator and the study team of the initiating study site. This group will meet once a week with study staff to review the progress of the study and ensure adherence to the study protocol. They will provide day-to-day support for the trial. The principal investigator will work together with the study team to resolve possible issues relating to study recruitment. On top of that, meetings with all co-investigators will be conducted once a month to discuss study progress.

The steering committee will provide oversight with monthly status reports that are shared with the principal investigator.

### Composition of the data monitoring committee, its role and reporting structure {21a}

Safety of the trial participants will be ensured by the institutional review board and the study team as they monitor the ethical conduct of this study, ensuring that the trial is implemented according to the protocol and that data are collected appropriately.

### Adverse event reporting and harms {22}

After participants have provided consent and been enrolled in the study, adverse events will be documented and recorded until the end of the study period. Any serious adverse event that occurs due to intervention or evaluation will be reported to the institutional review board and the research team of this study will take responsibility for the treatment.

### Frequency and plans for auditing trial conduct {23}

An annual audit will be conducted to protect the integrity of all collected data and study procedures as part of this trial. The principal investigator will ensure that data management is conducted and reported according to the protocol. The Project Management Group will meet weekly to review trial conduct through the study period. Annual auditing trials are planned to ensure the study protocol is followed.

A Data Monitoring Committee was not considered for this study as the intervention of this trial is considered as low risk.

### Plans for communicating important protocol amendments to relevant parties (e.g. trial participants, ethical committees) {25}

Any important protocol amendments will be communicated to the initiating study site (sponsor) and funder at first. The principal investigator will then notify the study centers. A copy of the revised protocol will be sent to the principal investigator and will be added to the Investigator Site File.

Protocol modifications will be updated as they occur in ClinicalTrials.gov. Documentation will be provided to study sites for their local review and implementation as required.

Furthermore, any deviations from the study protocol will be fully documented using a breach report form.

## Dissemination plans {31a}

Results of this trial will be available through scientific publications in a peer-reviewed journal and at scientific conferences. No other data sharing arrangements are intended.

## Discussion

We have described our planned protocol for a prospective, multicenter, randomized trial evaluating the influence of PNS on patients with chronic sacroiliac joint syndrome. We believe that PNS on patients with chronic sacroiliac joint syndrome will show promising results regarding pain relief and quality of life in comparison to BMT after 12 months. Clinical data on PNS for SIJP is still lacking and only limited retrospective data, technical notes and case reports have been published. Therefore, our main goal is to generate relevant evidence concerning the effect of PNS on patients with chronic intractable SIJP. The prospective, multicenter design of this trial promises high evidence in comparison to the data to date.

## Trial status

Recruitment started on 26 April 2022 and is still ongoing. The latest protocol version is 2.0 06 May 2022. Recruitment. The estimated primary completion date is April 2024.

## Data Availability

To promote study transparency, datasets and statistical analysis of this trial will be available from the corresponding author on reasonable request following completion of the trial.

## Abbreviations

SIJP: Sacroiliac joint pain
SCS: Spinal cord stimulation
PNS: Peripheral nerve stimulation
CTO: Clinical Trial Office
NRS: Numeric Pain Rating Scale
IPG: Implantable Pulse Generators
